# Antibiotic resistance mediated by gene amplifications

**DOI:** 10.1038/s44259-024-00052-5

**Published:** 2024-11-06

**Authors:** Kalinga Pavan T. Silva, Anupama Khare

**Affiliations:** Laboratory of Molecular Biology, National Cancer Institute, National Institutes of Health, Bethesda, MD 20892 USA

**Keywords:** Antimicrobial resistance, Antibiotics

## Abstract

Apart from horizontal gene transfer and sequence-altering mutational events, antibiotic resistance can emerge due to the formation of tandem repeats of genomic regions. This phenomenon, also known as gene amplification, has been implicated in antibiotic resistance in both laboratory and clinical scenarios, where the evolution of resistance via amplifications can affect treatment efficacy. Antibiotic resistance mediated by gene amplifications is unstable and consequently can be difficult to detect, due to amplification loss in the absence of the selective pressure of the antibiotic. Further, due to variable copy numbers in a population, amplifications result in heteroresistance, where only a subpopulation is resistant to an antibiotic. While gene amplifications typically lead to resistance by increasing the expression of resistance determinants due to the higher copy number, the underlying mechanisms of resistance are diverse. In this review article, we describe the various pathways by which gene amplifications cause antibiotic resistance, from efflux and modification of the antibiotic, to target modification and bypass. We also discuss how gene amplifications can engender resistance by alternate mutational outcomes such as altered regulation and protein structure, in addition to just an increase in copy number and expression. Understanding how amplifications contribute to bacterial survival following antibiotic exposure is critical to counter their role in the rise of antimicrobial resistance.

## Introduction

Antibiotic resistance is a major burden to public health as it leads to high infection-related hospitalizations, fatalities, and healthcare costs^1–3^. The misuse and overuse of antibiotics has been associated with the emergence of, and increase in, resistance^1,2^. Antimicrobial resistance can arise either via horizontal gene transfer events such as transformation, transduction, and conjugation, that propagate resistance cassettes between different bacterial strains and species, or through de novo mutations that are transmitted vertically^4,5^. Mutations underlying resistance are frequently single nucleotide polymorphisms, as well as small insertions and deletions. In addition, mutations like gene duplications and subsequent amplifications that increase the copy number of one or more genes can also drive the emergence of antibiotic resistance but are relatively understudied^6–8^.

**Fig. 1:**
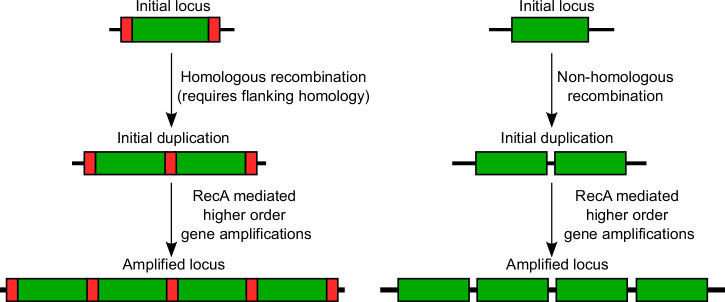
The formation of amplifications is a two-step process. In the first step, a region of the genome (green rectangles) is duplicated, either by (left) homologous recombination between flanking repeat sequences (red segments) typically mediated by RecA (although alternate mechanisms may also play a role)^9^, or (right) via non-homologous recombination. Due to the resulting homology, RecA-mediated homologous recombination can then generate additional copies leading to tandem gene amplifications.

The formation of amplifications is thought to be a two-step process, where the first step is a duplication of the region, followed by further amplification in the second step^9^ (Fig. 1). Initial duplications can be formed by homologous or non-homologous recombination events^8^. Homologous recombination is mediated by RecA, and requires flanking homology from either repeat sequences or transposable elements^9^. However, some duplications of regions between homologous ribosomal RNA loci (*rrn)* are thought to be mediated by single-strand annealing of the repeat regions, independent of RecA-mediated homologous recombination^9,10^. While all the mechanisms by which non-homologous recombination may result in duplications are not known, these likely include strand slippage or gyrase-mediated DNA break and ligation, as described in previous reviews on the topic^8,9^. As an abundance of homology is present after the initial duplication, subsequent amplification can be initiated by RecA-based homologous recombination, and the loss of amplification is also thought to be mediated by RecA^8,9^. Previous studies have shown that gene amplifications are heavily attenuated in a RecA mutant^9,11^.

It has been estimated that in *S*. Typhimurium, gene duplications may occur at some location in the genome in nearly 10% of cells in unselected culture^7,12^. Further, duplication frequencies at individual loci range from ~10^−2^ to ~10^−6^, with loci between repetitive *rrn* loci duplicating at much higher frequencies than regions not flanked by repetitive sequences^7,10,12^. In cases where duplications primarily result from RecA-mediated homologous recombination, the frequency of duplication is reduced 10-100-fold in mutants lacking RecA^9,13^. The frequency of subsequent amplification is influenced by rates of expansion as well as loss of amplifications. Spontaneous mutation frequencies in bacteria are estimated to be of the order of ~10^−10^ mutations per nucleotide per generation^14–16^, indicating that amplifications typically arise at much higher rates than specific mutations, and may thus play an important role in adaptation.

Gene amplifications are seen in organisms ranging from bacteria and yeast to plants, and mammals including in cancer cells^8,17–23^. Amplifications are associated with various adaptations in these systems, including resistance to carbon limitation, heavy metals, chemotherapeutic drugs, herbicides, and high temperatures, as well as growth on suboptimal carbon sources, overcoming auxotrophy, and even industrial overproduction of antibiotics^7,17,20,21,24–29^. Specifically, in bacteria, gene amplifications have been implicated in antibiotic resistance in laboratory selections, typically upon exposure to stepwise increases in antibiotic concentrations, as well as in clinical isolates^30–33^.

Here, we discuss the properties of antibiotic resistance conferred by gene amplifications, as well as the evolutionary trajectories and distinct mechanisms underlying such resistance. We focus mainly on recent studies, as older studies have been thoroughly discussed in previous reviews^7,8^.

## Properties of gene amplification-mediated antibiotic resistance

The tandem repeats present in amplifications typically make them unstable. Given this instability, antibiotic resistance due to amplifications has some properties distinct from that caused by more stable sequence-altering mutations.

**Fig. 2:**
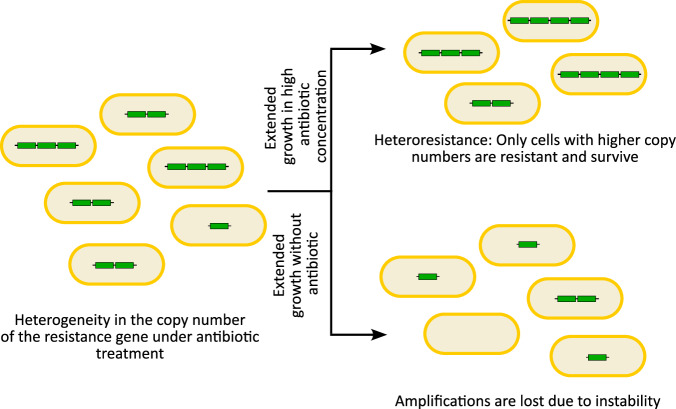
Gene amplifications lead to heteroresistance. Gene amplifications typically result in copy number variability of the amplified region in cells within a population when exposed to an antibiotic. Subsequent extended growth in high concentrations of the antibiotic will allow only the subpopulation with higher copy numbers to survive and grow, leading to heteroresistance, while extended antibiotic-free growth can result in instability and loss of the amplifications, reducing any potential fitness costs.

A typical feature of gene amplifications is heterogeneity in the copy number of the amplified locus within a population, resulting from contraction and expansion of the unstable amplification^8^ (Fig. 2). As a result, the population comprises cells with a high, moderate, or low number of, and possibly even zero, copies of the amplified gene. In case of antibiotic resistance, such variation in copy number leads to heteroresistance, where a sub-population of cells within an isogenic population displays resistance to an antibiotic, while the remainder of the population remains susceptible^34^. Heteroresistance has been seen in lab evolution experiments^33^, as well as in clinical isolates from a variety of pathogens including *Escherichia coli*, *Klebsiella pneumoniae*, *Salmonella enterica* serovar Typhimurium (*S*. Typhimurium), *Acinetobacter baumannii*, and *Enterobacter cloacae*^30,31^. It is thought that heteroresistance can lead to inaccurate or incongruent antibiotic susceptibility testing, treatment failure, as well as the emergence of resistance^34–36^. Characterizing the mechanisms underlying gene amplification events is therefore important to understand this clinically relevant process and can reveal strategies for treatment and prevention.

Due to the unstable nature of amplifications, alleviating the selective pressure (e.g. by passaging in selection-free media) typically leads to the reversion of amplifications, and thus increased susceptibility^8,33^ (Fig. 2). The reduction in copy number occurs both due to the inherent instability of the amplification due to tandem stretches of homologous sequences, and the potential fitness costs attributed to having multiple copies of genes^8^. Gene amplifications thus provide bacteria a dynamic reversible evolutionary path to survive in selective environments, and then rapidly reduce any fitness costs due to the amplification under non-selective conditions, unlike stable mutations^37^.

## Mechanisms of antibiotic resistance via gene amplifications

Mechanisms leading to antibiotic resistance include reducing the intracellular antibiotic concentration via increased efflux or lower cell permeability, inactivation of the antibiotic itself, antibiotic target modification or protection, or antibiotic activity bypass due to variant low-susceptibility targets, altered metabolism, or antibiotic sequestration^38^. While these are frequently mediated by sequence-altering mutations and acquisition of resistance determinants via horizontal gene transfer, gene amplification events can also lead to resistance through diverse mechanisms, as we discuss below. The examples we describe are summarized in Table 1. Additional instances of amplifications leading to antibiotic resistance have been detailed in previous reviews^7,34^.

**Table 1:** Examples of antibiotic resistance mediated by gene amplifications

Mechanism	Species	Antibiotic	Resistance gene	Source of strain	Reference
Decreased intracellular antibiotic concentration	*Streptococcus pneumoniae*	Ciprofloxacin, Norfloxacin, Levofloxacin	*patAB*	Lab	^48^
	*Staphylococcus aureus*	Ciprofloxacin	*norA*	Lab	^50^
	*S. aureus*	Delafloxacin	*sdrM*	Lab	^33^
	*Escherichia coli*	GP-6	*mdtK*	Lab	^62^
	*Acinetobacter baumannii*	GP-6	*mdtK*	Lab	^62^
Antibiotic inactivation	*A. baumannii*	Tobramycin	*aphA1*	Clinic and Lab	^70^
	*Enterobacter cloacae*	Cefiderocol	*bla*_SHV-5_	Clinic and Lab	^30^
	*Klebsiella pneumoniae*	Cefiderocol	*bla*_*NDM-5*_	Clinic	^30^
	*K. pneumoniae*	Amoxicillin	*bla*_SHV-11_	Clinic	^71^
	*Yersinia enterocolitica*	Ampicillin	*blaA*	Lab	^72^
	*E. coli*	Piperacillin-tazobactam	*bla*_*TEM-1*_	Lab	^73^
Target modification	*Salmonella* Typhimurium	Colistin	*pmrD, arnBCADTEF*	Lab	^79^
	*E. coli*	Polymyxin	*pmrD, arnBCADTEF*	Lab	^82^
	*S. aureus*	Vancomycin, Daptomycin	*mprF*	Lab	^83^
	*Enterococcus faecalis*	Macrolides	*ermA*	Lab	^93^
	*S. aureus*	Macrolides	*erm*(A)	Lab	^94^
	*S. pneumoniae*	Macrolides	L22 ribosomal protein	Clinic	^95^
Target bypass	*S*. Typhimurium	Albicidin	STM3175	Lab	^32^
	*E. coli*	Albicidin	*ygiV*	Lab	^32^
	*Streptococcus agalactiae*	Sulfonamide, trimethoprim	*folCEPBK*	Lab	^97^
	*S. aureus*	Oxacillin	SCC*mec*	Lab	^100^

### Decreased intracellular antibiotic concentration

Antibiotic resistance frequently develops due to mutations associated with efflux pumps. Generally, resistance mutations occur in the promoter regions of efflux pump encoding genes, thereby increasing efflux pump production^39,40^. Alternatively, inactivating mutations in transcriptional repressors can result in similar increases in expression of the pump^41,42^. Higher levels of efflux pumps can lead to the reduction of antibiotic concentrations inside the cell. Additionally, coding sequence mutations in efflux pumps can also lead to increased efflux activity by improving binding to a given antibiotic and thereby expelling it out of the cell more effectively^43,44^. Since efflux pump-mediated antibiotic resistance most often requires increased expression, this resistance mechanism is easily accessible via gene amplifications and has been seen in a variety of different species, especially for resistance against fluoroquinolone antibiotics.

In *Streptococcus pneumoniae*, the PatAB ABC transporter is associated with intrinsic fluoroquinolone resistance, and overexpression of the *patA* and *patB* genes in clinical isolates leads to resistance against ciprofloxacin (CPX)^45^. This overexpression can be induced by mutations in an upstream transcriptional terminator that are predicted to destabilize the terminator stem-loop to varying degrees, causing increased transcriptional read-through into the *patA* gene^46^. Further, in lab strains selected for resistance to the efflux pump inhibitor reserpine, *patAB* overexpression led to multidrug resistance against the fluoroquinolones CPX, norfloxacin, and levofloxacin, dyes such as acriflavine and ethidium bromide, and the biocide cetrimide^47^. This overexpression was caused by a novel duplication of a 9.2 kb region that included *patAB*^48^. The high levels of PatAB were not only due to the duplication itself, but also the transcriptional readthrough from a tRNA gene that was located upstream of the duplicated copy of the *patAB* gene, indicating that amplifications can increase expression not only via a higher copy number but also due to altered genomic context leading to a change in regulation.

In several species, specific clinical lineages are associated with high levels of antibiotic resistance, raising the possibility that they may have an increased propensity for the evolution of resistance^49^. Indeed, the experimental evolution of 222 diverse clinical isolates of *Staphylococcus aureus* in the presence of the fluoroquinolone CPX identified a wide range of evolvability of CPX resistance within these isolates^50^. Most strains evolved resistance via mutations in DNA topoisomerase IV, the primary target of many fluoroquinolones in most gram-positive bacteria, including *S. aureus*^51–53^. Interestingly, strains from clonal complex CC398 that showed high evolvability, evolved resistance via gene amplifications that elevated the copy number of a major facilitator superfamily (MFS) efflux pump *norA* 3-24-fold^50^. The *norA* amplifications were caused by two homologous copies of an IS (insertion sequence) element flanking the *norA* gene. IS elements are small transposable stretches of DNA that contain a transposase gene in between terminal inverted repeats^54^. The amplification of *norA* was not due to the transposition of the ISSau1 element to different genomic regions, but rather via tandem amplification due to the homologous copies of ISSau1^50^. This mechanism of resistance evolution was restricted to the CC398 lineage potentially due to the lineage-specific location of the ISSau1 transposons flanking the *norA* gene. Access to this homologous recombination-mediated tandem amplification-based mechanism of resistance likely resulted in the increased frequency of resistance evolution in this lineage compared to other lineages that acquired resistance via specific SNPs.

Multitargeting antibiotics often require multiple target mutations for resistance and are therefore thought to lead to lower frequencies of endogenous resistance development^55,56^. Delafloxacin (DLX) is a dual-targeting fluoroquinolone antibiotic that has similar affinities for both the DNA gyrase and topoisomerase IV enzymes and was recently approved for clinical use^57–60^. Experimental evolution of 10 independent populations of methicillin-resistant *S. aureus* (MRSA) in increasing concentrations of DLX revealed prevalent gene amplifications of a poorly characterized MFS efflux pump, *sdrM*, that led to high DLX resistance^33^. The evolved populations had 13 distinct gene amplifications 7-13 kb in size, and the junctions between the amplified segments had either microhomology or no homology. Interestingly, one end of the amplification was located in an rRNA-tRNA cluster, and consequently amplified copies of *sdrM* were located downstream of these highly expressed loci. While the copy number of the amplification in evolved isolates ranged from 3-10, the expression level of *sdrM* was increased 10-500-fold, possibly due to the upstream rRNA-tRNA cluster, similar to the *patAB* amplification seen in *S. pneumoniae*^48^. In the absence of *sdrM*, the evolution of DLX resistance occurred at a lower frequency and was mediated by mutations in both the targets, DNA gyrase, and topoisomerase IV, indicating that non-homologous recombination-mediated amplifications likely occur at a higher frequency than dual target coding sequence mutations^33^. Further, hitchhiking of two additional efflux pumps, *lmrS* and *sepA*, located adjacent to *sdrM*, in the amplifications led to cross-resistance against streptomycin.

**Fig. 3:**
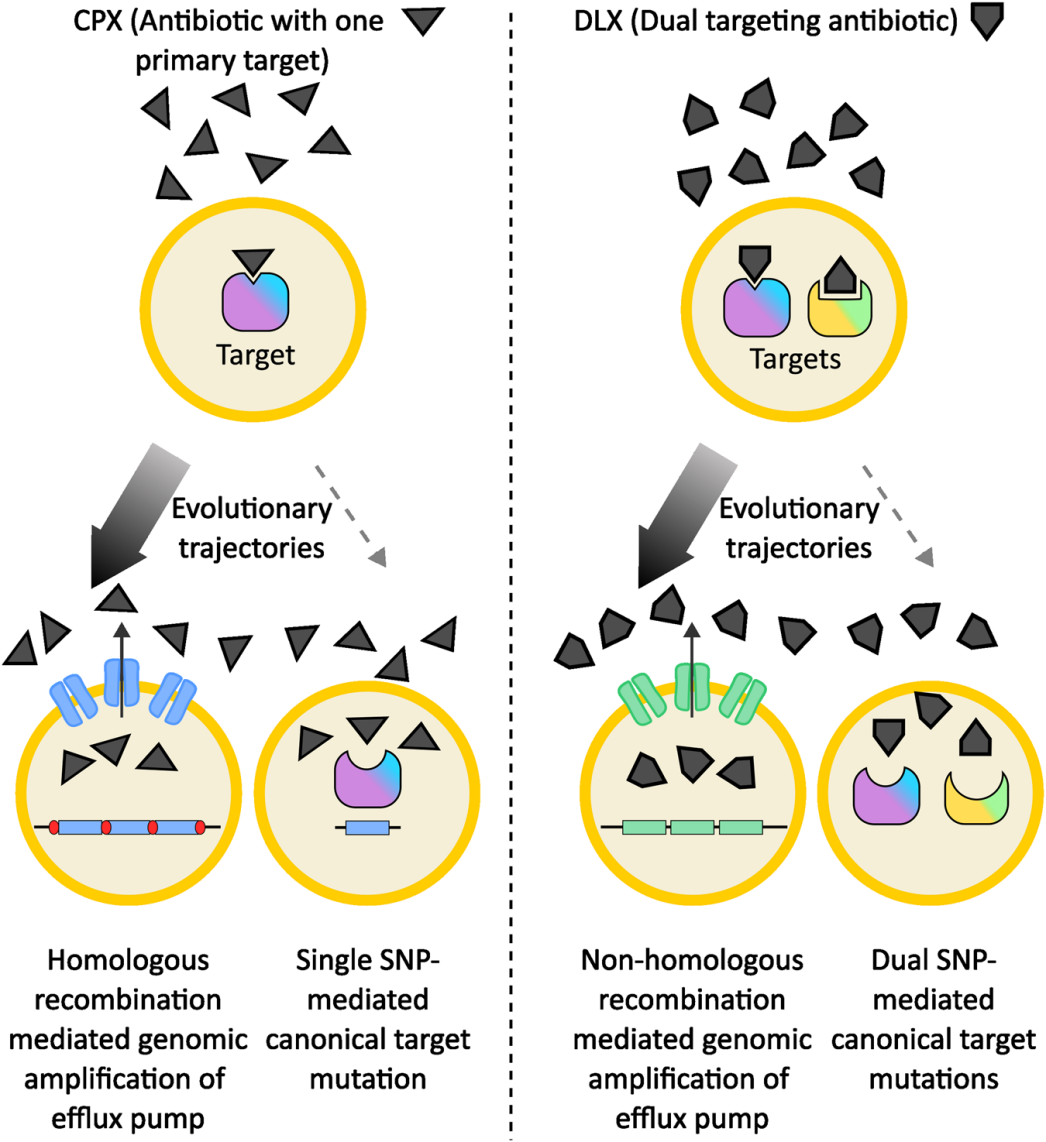
Gene amplifications often provide high-frequency evolutionary trajectories. In *S. aureus*, exposure to the antibiotic ciprofloxacin (CPX) that primarily targets the topoisomerase IV enzyme can select for resistance either via topoisomerase IV mutations or due to gene amplification of the efflux pump-encoding *norA* gene (blue rectangle). When *norA* is flanked by two identical transposable elements (red segments), homologous-recombination mediated gene amplifications provide the high-frequency evolutionary path (denoted by the thicker arrow). Similarly, exposure to the dual-targeting antibiotic delafloxacin (DLX) selects for resistance either via dual topoisomerase IV and gyrase mutations or due to the more frequent non-homologous recombination mediated amplification of the efflux pump-encoding *sdrM* gene (green rectangle). Thus, in both cases, gene amplifications provide the more common evolutionary trajectory instead of single or dual target mutations respectively.

It has been suggested that regions flanked by homologous sequences undergo gene duplication and amplification at a higher rate than those without surrounding homology^12,61^. In the above studies, amplifications mediated by homologous sequences, as seen in the IsSau1 mediated *norA* amplification, were more frequent than specific single nucleotide changes^50^, while amplifications between non-homologous regions, as seen in the *sdrM* amplifications, were more frequent than cells gaining two specific single nucleotide changes^33^, raising the possibility that this may be a general phenomenon where amplifications often provide more frequent paths to resistance evolution (Fig. 3).

A recent study of a different dual-targeting antibiotic provides another example of this phenomenon^62^. A set of two parallel studies compared evolutionary trajectories and genetic determinants of resistance in the gram-negative bacteria *E. coli* and *A. baumannii* against two different drugs – CPX^63^, which preferentially targets the DNA gyrase in most gram-negative species and the topoisomerase IV in most gram positive bacteria^52,53^, and the dual-targeting GP-6 compound belonging to the family of tricyclic GyrB/ParE (TriBE) inhibitors that target both the DNA gyrase and topoisomerase IV enzymes^62,64^. While target mutations in the gyrase enzyme were seen early and ubiquitously across all populations upon CPX exposure, such mutations were much less common upon GP-6 exposure and were preceded by mutations affecting drug efflux. For both species, five out of the six independently evolved populations showed gene amplification of the chromosomal *mdtK* gene that encodes an efflux pump belonging to the MATE (Multidrug And Toxic Compound Extrusion) family upon GP-6 exposure. Such gene amplification events were not seen in the presence of CPX, suggesting that similar to the more frequent amplification of *sdrM* in *S. aureus* upon DLX exposure^33^, the dual-targeting nature of GP-6 may have preferentially selected for gene amplifications^62^. However, given that GP-6 belongs to a different class of inhibitors compared to the fluoroquinolone CPX, additional drug characteristics may also influence the selection of amplifications versus other types of mutations.

### Antibiotic inactivation

Bacteria are known to synthesize various enzymes that are capable of modifying antibiotics, thereby neutralizing drug activity. The main classes of bacterial enzymes that modify antibiotics are hydrolases, transferases, and redox enzymes^65,66^. β-lactamases, aminoglycoside-modifying enzymes, and monooxygenases are known examples of these modification enzyme classes respectively^65–67^. These enzymes can lead to resistance through mutations that increase enzyme activity^68^, overexpression via point mutations in regulatory sites^69^, or gene amplifications^7^.

In clinical isolates, gene amplifications are often associated with heteroresistance and even treatment failure. A broad survey of clinical isolates of *E. coli, S*. Typhimurium*, K. pneumoniae* and *A. baumannii* displaying heteroresistance against many distinct antibiotics, especially aminoglycosides and β-lactams, identified spontaneous duplications and amplifications of resistance genes coding for inactivating enzymes^31^.

In one study, increase in antibiotic resistance of *A. baumannii* isolates from wound infections was associated with treatment failure in a patient treated with tobramycin for 4 days, resulting in a change in the antibiotics used for therapy^70^. Two *A. baumannii* isolates from this patient showed increased resistance due to distinct gene amplifications that emerged at two chromosomal positions containing the aminoglycoside resistance gene *aphA1*. The *aphA1* gene was flanked by two copies of IS26 in the transposon Tn*6020* and located in an antibiotic resistance island AbaR28. Amplification was seen in the original location in one isolate, while in the other isolate, Tn*6020* showed replicative transposition to a different genomic location, followed by amplification. Similar amplifications of *aphA1* were also selected for in vitro in sensitive strains upon tobramycin exposure.

In a separate study, a clinical isolate of *E. cloacae* heteroresistant to the β-lactam cefiderocol, was found to contain a 9.5 kb chromosomal gene amplification that included the β-lactamase gene *bla*_SHV-5_^30^. Identical transposase genes flanking the region likely led to the homologous recombination-mediated initial gene duplication, which was present in the clinical isolate at very low frequencies prior to cefiderocol treatment but increased in copy number upon cefiderocol exposure. Infection of *Galleria* waxworm moth larvae with this *E. cloacae* isolate, and subsequent cefiderocol treatment selected for the resistant subpopulations that carried higher copy numbers of the *bla*_SHV-5_ gene. The copy number of the amplification depended on the fitness benefit of the amplification, where a *bla*_*SHV-5*_ gene carrying a point mutation (SHV-5 M69I), encoding a lower activity mutant version of the SHV-5 β-lactamase, showed higher copy numbers. The copy number also depended on the selective pressure, and increased at higher concentrations of the antibiotic, as well as when the antibiotic was paired with clavulanate, a β-lactamase inhibitor.

Similar increases in resistance due to gene amplification-mediated β-lactamase gene overexpression have been seen in a variety of species, in both clinical isolates as well as lab evolved strains. A gene amplification containing a β-lactamase was seen in a *K. pneumoniae* strain isolated from a patient who failed cefiderocol treatment, after exposure to the antibiotic^30^. In a different study, two *K. pneumoniae* strains isolated from the same patient one month apart (before and after a relapse) differed in their antibiotic susceptibility. The strain isolated later had 16-fold higher amoxicillin resistance, associated with duplication of a chromosomal β-lactamase gene *bla*_SHV-11_^71^. Laboratory evolution of a *Yersinia enterocolitica* strain in increasing concentrations of ampicillin yielded evolved strains with MICs 20-fold higher than the wild type, that had tandem amplifications of a 28-kbp region that carried the β-lactamase encoding *blaA* gene^72^. Gene amplifications can also occur on plasmids, as seen in an *E. coli* clinical isolate where resistance to the β-lactam/ β-lactamase inhibitor combination piperacillin-tazobactam was associated with amplification-mediated overexpression of the *bla*_*TEM-1*_ gene located on a large plasmid similar to the p1ESCUM / p1ECUMN plasmid, originally isolated in an *E. coli* Clonal Group A clinical urine isolate^73,74^.

### Target modification

Apart from mechanisms involving the drug, such as reducing the intracellular antibiotic concentration, or inactivating the antibiotic, resistance can also be acquired by alterations to the drug target, whereby the target is no longer susceptible to antibiotic action^38,75^. Gene amplifications can provide access to these mechanisms by increasing the expression of pathways that modify antibiotic targets.

Colistin, also known as polymyxin E, is generally considered as a last line antibiotic to treat infections caused by multi-drug resistant gram-negative bacteria such as *S*. Typhimurium^76,77^. Colistin, which is positively charged, binds to the negatively charged phosphates from the lipopolysaccharide (LPS) of the outer membrane of gram-negative bacteria, displacing the cations Ca^2+^ and Mg^2+^ from the LPS and thereby disrupting the integrity of the membrane eventually leading to cell death^78^. Resistance is often acquired by mutations in *pmrAB*, genes encoding a two-component system, where the overexpression of *pmrA* leads to the upregulation of *pmrC*, *pmrE*, and *arnBCADTEF*, which catalyze the addition of phosphoethanolamine (PEtN) and 4-amino-4-deoxy-L-arabinose (L-Ara4N) to LPS, thereby reducing the net negative charge of LPS and inhibiting the interaction between the antibiotic and the cell^79^. Activation of PmrA-regulated genes occurs through the activity of the small protein PmrD, and plasmid-based overexpression of *pmrD* can thus lead to polymyxin resistance^80,81^. In an evolution of *S*. Typhimurium conducted by serially passaging it for many generations in sub-MIC concentrations of colistin, a majority of resistant populations contained gene amplifications sized between 49-301 kb. Interestingly, the *pmrD* gene and the *arnBCADTEF* operon were common in all amplified regions, with *pmrD* overexpression likely playing a major role in resistance^79^.

Similarly, in *E. coli*, polymyxin resistance results from increased addition of Ara4N and PEtN to LPS, mediated by *arn*/*pmr* or *ept*/*pmr* genes respectively. A recent study showed that in *E. coli* B strains, which are frequently resistant to polymyxin unlike *E. coli* K-12, amplification of the *arn* operon via flanking IS*1* elements led to polymyxin resistance in about ~30% of isolates selected for polymyxin resistance. In Δ*eptA* strains, where PEtN modifications are blocked leaving Ara4N modifications as the main resistance route, *arn* operon amplifications contributed to resistance in ~90% of selected resistant strains^82^. The frequency of resistance was ~70-fold lower when the flanking IS*1* element on one side was deleted, likely due to the unavailability of the homologous recombination-mediated amplification pathway for resistance. Further, resistance frequency was ~100-fold lower in *E. coli* K-12, where the *arn* operon is not flanked by IS elements, reiterating the importance of this resistance mechanism.

Alterations to the charge of the cell membrane in gram-positive bacteria are also associated with resistance. MRSA strains isolated on different days from a persistent infection in a single patient treated extensively with antibiotics showed acquisition of low-level vancomycin resistance^83^. Several strains had 10-98 kb chromosomal gene duplications, all of which included the genes *parC* (DNA topoisomerase IV subunit) and *mprF*. MprF is the phospholipid synthase and flippase that moves positively charged lysyl-phosphatidylglycerol to the outer leaflet of the cell membrane, resulting in the repulsion of, and therefore resistance against, cationic antimicrobial peptides, daptomycin, and potentially vancomycin^84–88^. The amplifications were associated with increased resistance to vancomycin, and the loss of the amplification in one strain was shown to increase vancomycin sensitivity^83^.

While gene duplications and amplifications typically lead to overexpression by increasing the copy numbers of a gene, they can also have phenotypic effects by altering gene regulation or protein structure. Macrolide antibiotics like erythromycin act by inhibiting ribosomal function. Resistance against macrolides, as well as the similar families lincosamide and streptogramin B, is frequently caused by *erm* (erythromycin resistance methylase) genes that encode a methylase enzyme that methylates the 23S rRNA, thereby inhibiting target binding by the antibiotic^89,90^. The *erm* genes frequently have leader peptides, and are regulated by translational attenuation^91,92^. In two separate studies in *S. aureus* and *Enterococcus faecalis*, short (25 bp or 72 bp respectively) tandem duplications of the ribosome binding site of the methylase gene were associated with macrolide resistance as they likely relieved translational repression via modification of the mRNA secondary structure^93,94^. An alternate mechanism of macrolide resistance involving a tandem duplication was seen in an *S. pneumoniae* clinical isolate that emerged during antibiotic therapy and led to treatment failure and a fatal relapse^95^. The resistant isolate did not have *erm* but had an 18 bp tandem duplication in the gene encoding the ribosomal protein L22. The resulting six amino acid duplication in this target ribosomal protein likely inhibited the binding of the drug to its target, thereby leading to resistance.

### Target bypass

Antibiotic resistance can also arise via mechanisms that allow for target bypass^38^. These include increased expression of the antibiotic target, antibiotic sequestering proteins, proteins that perform the same function as the antibiotic target but are not susceptible to the antibiotic target, or metabolic pathways that override the action of the antibiotic.

The antibacterial peptide, albicidin, inhibits the DNA gyrase enzyme, and resistance to albicidin in *E. coli* is frequently associated with mutations in a transporter Tsx that block albicidin transport across the outer membrane into the cell^96^. However, one wild-type population of *S*. Typhimurium subjected to a high concentration of albicidin and several Δ*tsx* populations subjected to increasing concentrations of albicidin yielded evolved mutants that had gene amplifications of sizes ranging from 3kb-158kb and an 80-1000-fold increase in MIC^32^. All the gene amplifications contained a putative regulatory protein STM3175, which contains a helix-turn-helix DNA binding domain as well as a GyrI-like ligand binding domain. This ligand binding domain likely binds to albicidin and sequesters it, thereby leading to resistance. Homologs of STM3175 are present in *E. coli*, *Vibrio vulnificus*, and *Pseudomonas aeruginosa* and the overexpression of these homologs also led to albicidin resistance. Interestingly, when *E. coli* was evolved with increasing concentrations of albicidin, several evolved strains carried gene amplifications containing the homolog of the STM3175-encoding gene, *ygiV*, indicating that overexpression of this albicidin-binding protein via gene amplifications may be a common evolutionary trajectory in multiple pathogens upon albicidin exposure.

The antibiotics sulfonamide and trimethoprim each act on different enzymes of the folate biosynthesis pathway. A *Streptococcus agalactiae* strain carrying an amplification that included five genes involved in folate biosynthesis, *folCEPBK*, was resistant to both these antibiotics^97^. Sulfonamide resistance likely resulted from an overproduction of its target enzyme dihydropteroate synthase, encoded by *folP*, a mechanism distinct from target modifications or horizontal acquisition of insensitive targets that are commonly seen in diverse species^98^. Trimethoprim resistance is similarly associated with modification of the chromosomal target enzyme dihydrofolate reductase (DHFR) or gain of plasmids carrying trimethoprim-insensitive DHFR enzymes. In contrast, in this study, amplification of the folate biosynthesis pathway was expected to lead to the accumulation of its product dihydrofolate, the substrate of DHFR^97^. Increased substrate concentration likely allowed for increased DHFR function, thereby overcoming trimethoprim inhibition and leading to resistance. Thus, a single gene amplification can lead to cross-resistance by two different mechanisms – production of excess of either the target enzyme or the substrate of the target enzyme.

MRSA strains are resistant to β-lactams as they carry the *mecA* gene encoding an alternate penicillin-binding protein (PBP) with low affinity to β-lactams on a mobile genetic element called the staphylococcal cassette chromosome (SCC*mec*)^99^. A MRSA strain evolved in a chemostat for 13 days under increasing concentrations of the β-lactam oxacillin yielded hyper-resistant strains^100^. In one of these strains, the copy number of the SCC*mec* element was elevated 10-fold via tandem gene amplifications. Despite being unstable in the absence of antibiotics, this hyper-resistant strain outcompeted the parental strain in the presence of sub-inhibitory concentrations of oxacillin. Thus, amplification of an alternate low-susceptibility antibiotic target can lead to high-level resistance.

## Gene amplifications associated with antibiotic resistance can lead to fitness alterations

Canonical resistance mutations often cause fitness defects and are therefore frequently deleterious when the selective pressure of the antibiotic is removed^101^. In some cases, gene amplifications can counter such fitness disadvantages, thereby promoting the survival and potential spread of antibiotic resistance. Additionally, gene amplifications associated with antibiotic resistance can have unexpected unrelated fitness consequences that may alter bacterial survival under other stresses.

Bacterial translation is typically initiated with a formyl Met-tRNAi, and after translation is complete, the formyl group is removed by a peptide deformylase enzyme, allowing the full-length peptide to mature correctly^102^. Actinonin belongs to a class of antibiotics that inhibit the peptide deformylase enzyme. *S*. Typhimurium isolates that were resistant to actinonin were found to have likely loss-of-function mutations in *fmt*, a gene that encodes for methionyl-tRNA formyltransferase which adds a formyl group to the Met-tRNAi, or *folD*, which encodes an enzyme required for the biosynthesis of formylated Met-tRNAi precursors. Without either of these enzymes, unformylated Met-tRNA is utilized for protein production, obviating the need for peptide deformylase activity. However, *fmt* and *folD* mutations lead to high fitness costs both in vitro and in vivo, with reduced growth rates, likely due to impaired translation^102,103^. By serially passaging mutant isolates for 50-150 generations in a rich medium, evolved isolates showed compensation for the growth defect. Several of these mutants that retained actinonin resistance contained gene amplifications of *metZ* and *metW*, that encode tRNAi. The elevated copy number of *metZW* results in an abundance of tRNAi, and this high concentration of unformylated Met-tRNAi can likely overcome the inefficient translation initiation rate in these resistant mutants.

Similar translation-related gene amplifications leading to increased fitness have been seen with a different antibiotic in *S*. Typhimurium. The antibiotic mupirocin is an analog of isoleucyl–adenylate, which enables it to bind to isoleucyl–tRNA synthetases (IleRS) thereby attenuating the transfer of isoleucine to its cognate tRNA molecule. In *S*. Typhimurium, resistance against mupirocin can be acquired by mutations in the gene *ileS*, encoding the IleRS enzyme, likely by reducing binding to mupirocin or altering binding to isoleucine^104^. However, these mutations often lead to severe fitness defects in the absence of the antibiotic due to inefficient IleRS activity^104^. Subsequent passaging of such a resistant strain in antibiotic-free media yielded several mutants that carried gene amplifications of *ileS*, and the increased expression of the defective IleRS enzyme led to the recovery of fitness^105^.

Selection of antibiotic resistance may also inadvertently select for amplifications that have unforeseen fitness consequences. Growth of an *E. cloacae* strain in colistin resulted in a 9-kb gene amplification that included the nickel resistance operon *ncrABC*. This gene amplification did not lead to colistin resistance but instead led to a 4-fold increase in nickel resistance^106^. The amplified segment was flanked by transposable elements, which likely led to the initiation of the amplification. While the selective pressures underlying the selection of this amplification in the presence of colistin are unknown, this study shows that antibiotic exposure can unexpectedly lead to resistance against a secondary bactericidal treatment via gene amplifications.

## Conclusions

**Fig. 4:**
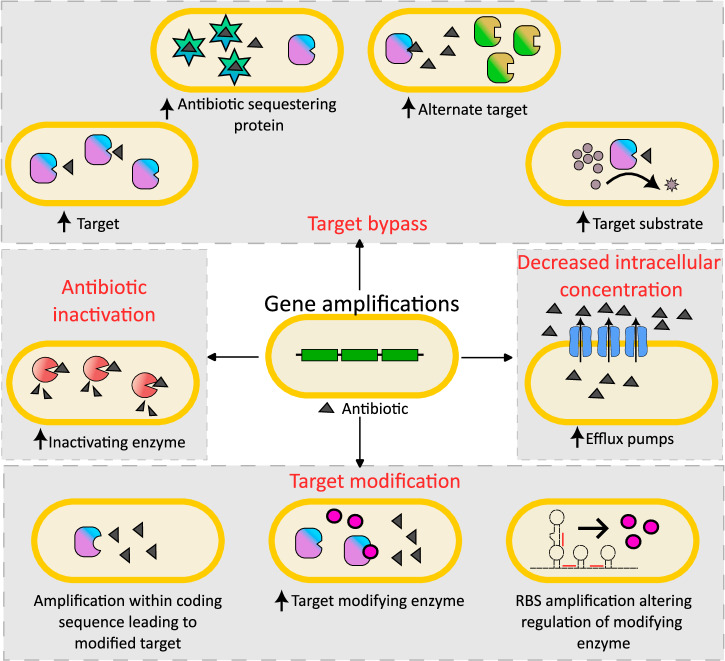
Mechanisms of antibiotic resistance via gene amplifications. Gene duplications and amplifications can lead to antibiotic resistance by a variety of mechanisms including increased drug efflux, drug inactivation via overexpression of inactivating enzymes, target modification by increased expression of modifying enzymes or modifying target structure, and target bypass via overexpression of the target itself, drug-sequestering proteins, alternate low-susceptibility targets, or substrates of the antibiotic target. While amplifications most often function by increasing expression through higher copy numbers, amplifications can also result in altered regulation or protein structure, leading to resistance.

Gene duplications and amplifications offer a viable but reversible avenue for bacterial cells to acquire antibiotic resistance. As described above, gene amplifications can lead to antibiotic resistance via diverse mechanisms (Fig. 4, Table 1), and fitness costs associated with gene amplifications can be ameliorated by the loss of amplifications once the selective pressure is removed. However, this dynamic nature of amplifications also makes them hard to detect. Additionally, the repetitive nature of amplifications makes these sequences more difficult to assemble and identify, especially with short-read sequencing, and their role in adaptation to antibiotics and other stresses is thus likely underappreciated. Despite this, as discussed above, gene amplifications have been identified in several independent studies from clinical samples in a wide variety of species, where they frequently lead to antibiotic resistance and have occasionally even been associated with treatment failure. Identifying mechanisms and pathways that underlie the formation of gene amplifications, especially in clinically relevant bug-drug combinations, can thus reveal putative novel targets for therapies that may improve treatment outcomes, and even reduce the frequency of the evolution of antibiotic resistance.

Amplifications are commonly selected under stress conditions, including exposure to DNA-damaging antibiotics such as fluoroquinolones as well as other antibiotics, inhibition of tRNA synthesis, and nutrient limitation^30,32,33,103,107^. The responses to these physiological challenges and alterations include the general stress response, as well as specific stress responses such as the SOS pathway and other DNA repair mechanisms, and what role these stress response pathways could potentially play in the generation and maintenance of amplifications, has not been exhaustively characterized.

Since RecA plays a role in generating amplifications, targeting RecA can be an attractive prospect as a therapeutic approach to limit resistance via amplifications. However, recent work has shown that the absence of *recA* can lead to rapid evolution of resistance^108^. Thus, further work is needed to delineate additional genes and mechanisms that contribute to the formation and loss of gene duplications and amplifications, to determine viable therapeutic targets.
